# RAD-140 Drug-Induced Liver Injury

**DOI:** 10.31486/toj.22.0005

**Published:** 2022

**Authors:** Kenneth Leung, Priyanka Yaramada, Preeya Goyal, Cindy X. Cai, Irene Thung, Muhammad B. Hammami

**Affiliations:** ^1^ Department of Gastroenterology and Hepatology, VA Loma Linda Healthcare System, Loma Linda, CA; ^2^ University of California, Riverside, Riverside, CA; ^3^ Loma Linda University, Loma Linda, CA

**Keywords:** *Chemical and drug induced liver injury*, *Enobosarm*, *LGD-4033*, *RAD140*

## Abstract

**Background:** RAD-140, one of the novel selective androgen receptor modulators (SARMs), has potent anabolic effects on bones and muscles with little androgenic effect. Despite the lack of approval for its clinical use, RAD-140 is readily accessible on the consumer market. Hepatotoxicity associated with the use of SARMs has only rarely been reported in the literature.

**Case Report:** A 24-year-old male presented with a 2-week history of diffuse abdominal pain, scleral icterus, pruritus, and jaundice. Prior to presentation, he had been taking the health supplement RAD-140 for muscle growth for 5 weeks. He had a cholestatic pattern of liver injury, with a peak total bilirubin of 38.5 mg/dL. Liver biopsy was supportive of a diagnosis of RAD-140–associated liver injury characterized pathologically by intracytoplasmic and canalicular cholestasis with minimal portal inflammation. Symptoms and liver injury resolved after cessation of the offending agent.

**Conclusion:** To date, only select descriptions of the potential hepatoxicity associated with the use of SARMs, including RAD-140, have been published. Given their potential hepatoxicity and ready availability on the consumer market, RAD-140 and other SARMs should be used judiciously and under close clinical supervision until further hepatic safety data become available.

## INTRODUCTION

Nonsteroidal selective androgen receptor modulators (SARMs), including RAD-140, Enobosarm, and Ligandrol, promote anabolic effects on bones and muscles through binding to androgen receptors, similar to traditional anabolic-androgenic steroids such as testosterone.^1-3^ Unlike anabolic-androgenic steroids, SARMs do not have the unwanted androgenic effects because they are not metabolized to dihydrotestosterone by 5-alpha reductase or to estrogen by aromatase.^2^ Because of the high tissue selectivity of their anabolic actions, SARMs are being studied in several clinical applications, including sarcopenia, cancer-related cachexia, prostate cancer, breast cancer, and osteoporosis.^1^ Despite receiving a warning from the US Food and Drug Administration and being banned by the World Anti-Doping Agency, SARMs remain readily accessible in the online marketplace and are used as alternatives to anabolic-androgenic steroids for muscle and strength development.^4-6^ Unlike anabolic-androgenic steroids, which have a well-known hepatotoxic profile, only limited hepatic safety data are available for SARMs.^3,7-12^ We report a case of drug-induced liver injury caused by the use of the SARM RAD-140.

## CASE REPORT

A previously healthy 24-year-old male presented to the emergency department (ED) with a 2-week history of diffuse abdominal pain, pruritus, scleral icterus, and jaundice. Approximately 2 months earlier, he had started taking up to 15 mg daily of RAD-140 for a total of 5 weeks for muscle growth. The only other medication that he took was acetaminophen 250 mg/aspirin 250 mg/caffeine 65 mg (used 4 times daily as needed for headache, no more than twice a week). He had stopped both medications 2 weeks prior to presentation as instructed by his primary care provider (PCP) because of the incidental finding of elevated liver chemistries (Table 1, Routine PCP office visit, +5 weeks).

**Table 1.** Liver Test Results

Timeline	Total Bilirubin, mg/dL (Reference, 0.2-1.2)	ALT, IU/L (Reference, 17-63)	AST, IU/L (Reference, 15-41)	ALP, IU/L (Reference, 32-91)	Platelet Count, × 10^9^/L (Reference, 150-450)	INR (Reference, <1.0)	Albumin, gm/dL (Reference, 3.5-4.8)
Prior to RAD-140 use, –14 months	1.0	48	26	82	152	–	4.6
Started RAD-140	–	–	–	–	–	–	
Routine PCP office visit, +5 weeks	1.2	313	182	103	180	0.8	4.4
Emergency department encounter, +7 weeks	10.8	171	71	151	263	0.85	4.1
Hepatology office visit, +8 weeks	32.3	125	82	181	305	0.96	3.9
Hepatology office visit, +10 weeks	24.8	212	120	251	310	1.47	3.0
Hospital day 1, +10 weeks, 2 days	28.7	155	94	232	341	1.36	2.9
Hospital day 2	26.1	134	76	203	312	1.46	2.5
Hospital day 3	27.3	115	62	199	260	1.61	2.6
Hospital day 4	24.3	117	79	194	268	1.69	2.5
Postdischarge hepatology office visit, +11 weeks	38.5	143	104	189	299	0.84	2.9
Postdischarge hepatology office visit, +14 weeks	24.6	120	118	187	261	0.89	2.9
Postdischarge hepatology office visit, +18 weeks	4.9	118	79	118	170	0.91	4.0

ALP, alkaline phosphatase; ALT, alanine transaminase; AST, aspartate aminotransferase; INR, international normalized ratio; PCP, primary care provider.

In the ED, the patient denied consuming alcohol or using any other supplements, over-the-counter or prescription medications, or recreational drugs. Physical examination was normal except for jaundice and scleral icterus. Laboratory data revealed total bilirubin of 10.8 mg/dL (reference range, 0.2-1.2 mg/dL), alkaline phosphatase of 151 IU/L (reference range, 32-91 IU/L), alanine aminotransferase of 171 IU/L (reference range, 17-63 IU/L), and aspartate aminotransferase of 71 IU/L (reference range, 15-41 IU/L). Gamma-glutamyl transpeptidase, international normalized ratio (INR), total protein, albumin, and complete blood count were within normal limits (Table 1, Emergency department encounter, +7 weeks). Hospital admission for further testing was recommended; however, the patient declined and left against medical advice.

A week after his index hospital encounter, the patient was seen in the hepatology clinic for follow-up, where he was noted to have worsening jaundice and pruritus. Blood work revealed total bilirubin of 32.3 mg/dL, alkaline phosphatase of 181 IU/L, alanine aminotransferase of 125 IU/L, aspartate aminotransferase of 82 IU/L, and INR of 0.96 (Table 1, Hepatology office visit, +8 weeks). Based on the patient's initial assessment, drug-induced liver injury from recent RAD-140 use was suspected. However, as he did not show any signs or symptoms concerning for acute liver failure that would warrant immediate hospitalization, further workup for his abnormal liver tests and short-interval follow-up were arranged. Two weeks later, he returned to the hepatology clinic, and blood work revealed total bilirubin of 24.8 mg/dL, alkaline phosphatase of 251 IU/L, alanine aminotransferase of 212 IU/L, aspartate aminotransferase of 120 IU/L, and INR of 1.47 (Table 1, Hepatology office visit, +10 weeks).

Given his significantly elevated liver tests and now elevated INR, he was urged to go to the ED, where he was admitted for closer monitoring and diagnostic evaluation. He did not display any signs or symptoms of encephalopathy. He denied any interval use of prescription or over-the-counter medications, supplements, alcohol, or recreational drugs. Serologic markers for viral hepatitis and autoimmune liver conditions were negative. Serum ferritin was significantly elevated at 1,523 ng/mL (reference range, 22-322 ng/mL), but transferrin saturation was only 21% (reference, ≤45%) and he was negative for C282Y/H63D mutations. Ceruloplasmin level was normal. Alpha-1 antitrypsin (AAT) level was normal, and AAT phenotype was Pi*MZ. Urine toxicology screen, alcohol screen, and acetaminophen level were negative. Doppler ultrasound of the liver was unremarkable. Abdominal axial computed tomography and magnetic retrograde cholangiogram revealed hepatomegaly and limited focal fatty infiltration with patent biliary tree and vasculature (Figure, A and B). Percutaneous liver biopsy revealed bile accumulation inside the hepatocytes and canaliculi (Figure, C) with minimal portal infiltrates of lymphocytes (Figure, D) and no significant steatosis, necrosis, fibrosis, stainable iron, or intracytoplasmic inclusions on periodic acid–Schiff stain, consistent with drug-induced cholestasis.

**Figure.**
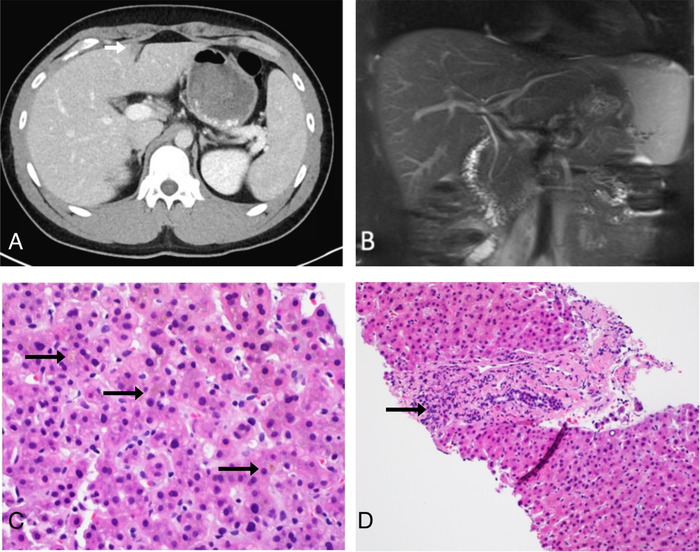
(A) Axial contrast-enhanced computed tomography image shows hepatomegaly and focal fatty infiltration (arrow). (B) Magnetic resonance cholangiopancreatography demonstrates patent biliary tree. (C) Liver histology illustrates hepatocellular and canalicular cholestasis (arrows) (hematoxylin and eosin [H&E] stain, magnification ×40). (D) Liver histology reveals minimal portal inflammation (arrow) (H&E stain, magnification ×20).

The patient had an unremarkable hospital course. Serial laboratory testing showed gradually improving liver chemistries and stable INR. As acute liver failure was not a concern, N-acetylcysteine was not given.

The patient was discharged on hospital day 4. He returned to the hepatology clinic for regular visits up to 5 months postdischarge. At his most recent clinic visit, his symptoms had entirely resolved, and his liver tests had markedly improved (Table 1, Postdischarge hepatology office visit, +18 weeks). The patient did not return for his next scheduled hepatology clinic visit and did not return calls to reschedule. Approximately 14 months postdischarge, he was seen at the PCP clinic for reasons unrelated to his liver injury. Routine blood work including liver tests was ordered but not completed.

## DISCUSSION

This case of RAD-140–associated cholestatic liver injury had an assessed Drug-Induced Liver Injury Network causality score of 1 (probable) and a severity score of 3 (severe injury).^8^ To our knowledge, ours is the sixth case of SARM-associated drug-induced liver injury and the third case associated with RAD-140 use (Table 2).^9-12^ Similar to the previously reported cases, jaundice was the presenting symptom in our patient. Except for one case, SARM-related liver injury was either of mixed or cholestatic pattern.^9-12^ In our case, we observed a primarily cholestatic pattern of injury, with an initial moderate elevation in transaminases, followed by profound hyperbilirubinemia. We observed a latency period of approximately 6 weeks from cessation of the SARM to peak bilirubin levels, compared to Flores et al who reported a latency period of 5 weeks.^9^ Similar to Flores et al and Bedi et al, we observed a mild elevation in INR without fulminant liver failure.^9,12^ Liver biopsies typically demonstrate intracytoplasmic or canalicular cholestasis with minimal or nonexistent necrosis or inflammation. In all cases of SARM-related hepatoxicity to date, including ours, liver injury resolved with cessation of the offending agent. No patients developed fulminant liver failure, and no deaths occurred. While our patient did not receive any specific treatment for his liver injury, ursodeoxycholic acid may be beneficial in relieving cholestasis related to drug-induced liver injury, although data are largely lacking.^13^

**Table 2.** Summary of Cases of Selective Androgen Receptor Modulator (SARM)-Associated Hepatotoxicity

Study	SARM	Presenting Symptoms	Duration of SARM Use	Initial / Peak Bilirubin, mg/dL	Time to Peak Bilirubin After SARM Cessation	Initial ALT / AST / ALP, IU/L	Initial Platelet Count, × 10^9^/L	INR	Liver Histology	Management
Flores et al, 2020^9^	Ligandrol	Jaundice, anorexia, nausea, lethargy, weight loss	9 weeks	6.78/6.78	5 weeks	273/111/289	387	1	–	SARM cessation, supportive care
Barbara et al, 2020^10^	Ligandrol	Jaundice, fatigue, pruritus, weight loss	2 weeks	35/38.2	–	229/91/88	–	1.1	Cholestatic hepatitis with mild portal, periportal, and perisinusoidal fibrosis	SARM cessation, supportive care
Bedi et al, 2021^12^	Enobosarm	Jaundice, anorexia, weight loss, lethargy, diarrhea	2 months	19.9/43.0	–	112/69/268	313	1.3	Moderate to severe cholestasis, very mild ductal damage	SARM cessation, supportive care
Flores et al, 2020^9^	RAD-140	Jaundice, pruritus	4 weeks and intermittent use thereafter	17/20.2	–	44/61/286	347	1.2	Moderate cholestasis, ductopenia, minimal fibrosis and inflammation	SARM cessation, supportive care
Barbara et al, 2020,^11^	RAD-140 / Ligandrol	Jaundice, right upper quadrant pain, pruritus, diarrhea	7 weeks	34.5/34.5	–	46/36/529	–	1.0	Diffuse canalicular cholestasis, ductal reaction, mild lobular inflammation with rare non-necrotizing epithelioid granuloma and mild portal and periportal fibrosis	SARM cessation, supportive care
Present case, 2022	RAD-140	Jaundice, abdominal pain, scleral icterus, pruritus	5 weeks	1.2/38.5	5 weeks, 6 days	313/182/103	180	0.8	Moderate intracytoplasmic and canalicular cholestasis, minimal portal inflammation	SARM cessation, supportive care

ALP, alkaline phosphatase; ALT, alanine transaminase; AST, aspartate aminotransferase; INR, international normalized ratio.

Our patient had a history of using acetaminophen and salicylic acid prior to the onset of his liver injury. However, acetaminophen and/or salicylic acid–related hepatoxicity was deemed unlikely because of the small accumulated dosage, and more importantly, the lack of classic zone 3 necrosis/apoptosis and/or microvesicular steatosis. On the other hand, bland cholestasis, a pathologic feature commonly observed in anabolic-androgenic steroid–induced hepatotoxicity, highly suggests cholestatic injury from RAD-140.^14^

The molecular mechanisms underlying SARM-induced hepatotoxicity are largely speculative. The bland cholestasis seen in both anabolic-androgenic steroid– and RAD-140/Enobosarm–associated hepatotoxicity highly suggests involvement of androgen receptors in dysregulation of bile transport. In animal studies, the bile salt export pump (BSEP), an ATP-binding cassette subfamily B member 11 (ABCB11) transporter, was reported to be involved in anabolic-androgenic steroid–induced cholestatic injury.^7,15,16^ Whether the association of RAD-140 with androgen receptors regulates BSEP through receptor-associated signaling pathways remains to be defined. In humans, the ABCB11 mutation was reported to increase the genetic susceptibility of anabolic-androgenic steroid–induced cholestasis.^17^ Although we do not know if our patient has the ABCB11 mutation, he is heterozygous for alpha-1-antitrypsin Z (Pi*MZ), a phenotype reported to be a predisposing factor for liver disease and fibrosis.^18^ Whether Z heterozygosity contributes to drug-induced cholestasis remains to be determined.

## CONCLUSION

The accumulating cases of drug-induced liver injury from SARMs raise concern about their hepatic safety and question the tissue selectivity of these agents. We caution the use of SARMs outside of clinical investigation and advocate for tighter regulation, close monitoring, and prompt reporting of adverse events associated with SARMs.

## Notes

*This article meets the Accreditation Council for Graduate Medical Education and the American Board of Medical Specialties Maintenance of Certification competencies for Patient Care and Medical Knowledge.*
